# treeman: an R package for efficient and intuitive manipulation of phylogenetic trees

**DOI:** 10.1186/s13104-016-2340-8

**Published:** 2017-01-07

**Authors:** Dominic J. Bennett, Mark D. Sutton, Samuel T. Turvey

**Affiliations:** 1 Department of Earth Science and Engineering, Imperial College London, London, UK; 2 Institute of Zoology, Zoological Society of London, London, UK

**Keywords:** Phylogenetic trees, Evolution, Tree simulation, R, Statistical computing

## Abstract

**Background:**

Phylogenetic trees are hierarchical structures used for representing the inter-relationships between biological entities. They are the most common tool for representing evolution and are essential to a range of fields across the life sciences. The manipulation of phylogenetic trees—in terms of adding or removing tips—is often performed by researchers not just for reasons of management but also for performing simulations in order to understand the processes of evolution. Despite this, the most common programming language among biologists, R, has few class structures well suited to these tasks.

**Results:**

We present an R package that contains a new class, called TreeMan, for representing the phylogenetic tree. This class has a list structure allowing phylogenetic trees to be manipulated more efficiently. Computational running times are reduced because of the ready ability to vectorise and parallelise methods. Development is also improved due to fewer lines of code being required for performing manipulation processes.

**Conclusions:**

We present three use cases—pinning missing taxa to a supertree, simulating evolution with a tree-growth model and detecting significant phylogenetic turnover—that demonstrate the new package’s speed and simplicity.

**Electronic supplementary material:**

The online version of this article (doi:10.1186/s13104-016-2340-8) contains supplementary material, which is available to authorized users.

## Background

Phylogenetic trees have been a mainstay of the R statistical software environment since the release of Emmanuel Paradis’ APE package in 2002 [1, 2]. This package introduced the phylo object, an S3 class for the presentation and manipulation of phylogenetic tree data in the R environment. In its most basic implementation, the phylo object contains a list of three elements: an edge matrix, a vector of tip labels and an integer of the number of internal nodes. The use of an edge matrix facilitates phylogenetically structured statistical analyses because of its convenience for generating distance, cophenetic or covariance matrices. For this reason the APE package’s phylo is the dominant class for phylogenetic tree representation in R and is used by many well-known phylogenetic R packages (e.g. phangorn [3], phytools [4]). Since phylo’s first incarnation the number of available functions in the APE package has risen from 28 to 171 (versions 0.1–3.4), and to date there are 147 reverse dependencies, i.e. packages on CRAN [1] that depend on the phylo class. More recently, the phylo class has been updated to S4 as part of the phylobase package [5].

An edge matrix, however, leads to a dependence on index referencing, leading to certain computational scenarios in which the phylo object performs poorly: in particular, analyses that require the manipulation of the tree itself (i.e. tip and node addition/deletion). Such analyses include simulating, comparing, pruning, and merging trees, and calculating phylogenetic statistics such as measures of phylogenetic richness [6] and evolutionary distinctness [7]. These have become the preserve of software solutions external to R, e.g. [8, 9], hindering their integration with the many packages in biomolecular, evolutionary and ecological studies already available for R. Although there are alternatives to the phylo class for phylogenetics or more generally ‘networks’ available in R [10], these packages and classes are rarely used for phylogenetics and may lack the intuitive functional framework for manipulating evolutionary trees.

**Fig. 1:**
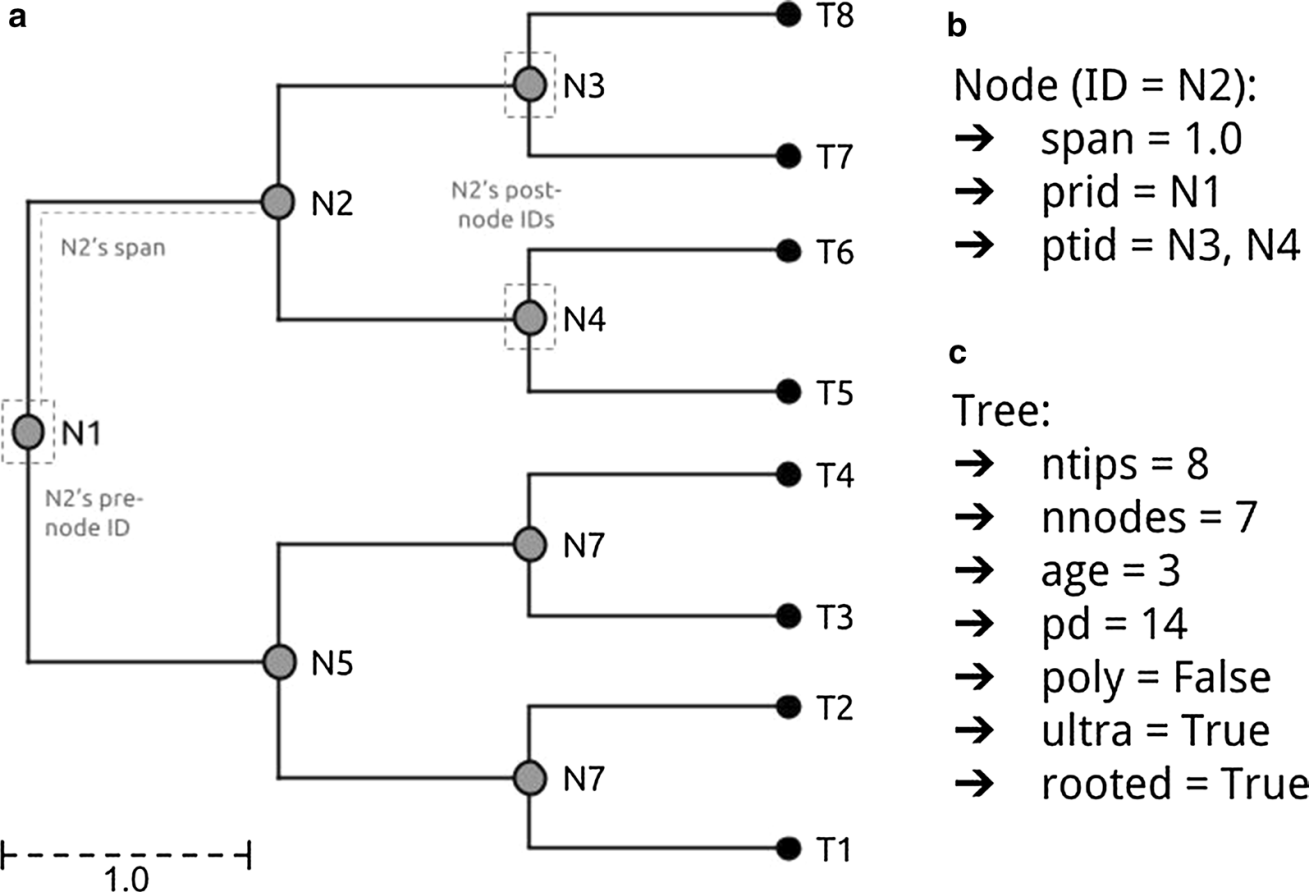
Representation of simple tree as TreeMan object: an eight-tipped tree with node and tip IDs annotated and N2′s key slots identified, **b** representation of printed Node information for N2, **c** representation of printed TreeMan information

**Fig. 2:**
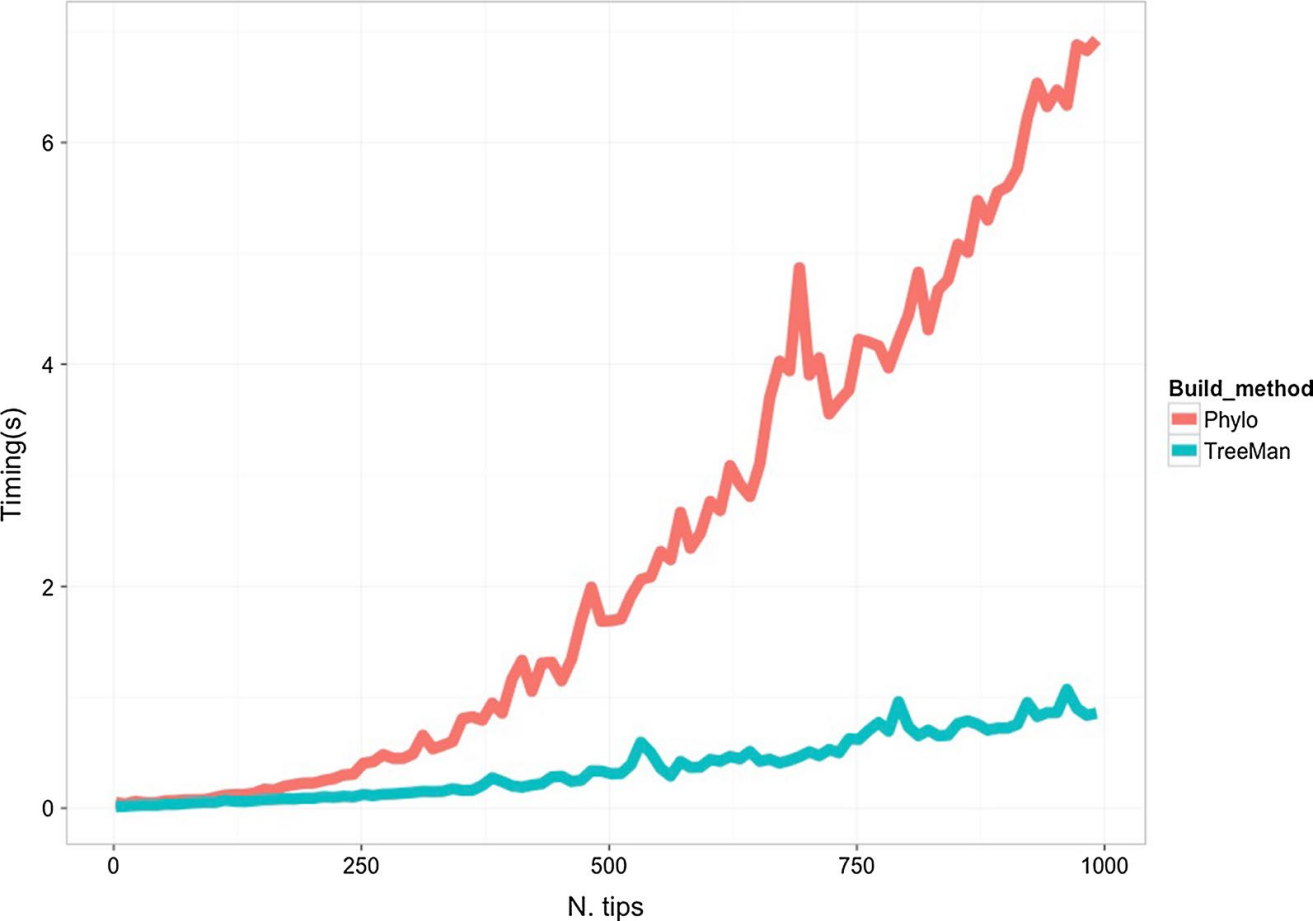
Comparison of tree building using APE’s phylo and treeman’s TreeMan classes. Starting with trees of two tips, 1000 new tips were added to the trees and the time taken to run the process was recorded every ten tips. The rate of increase in time taken for larger trees increases faster for the phylo class than TreeMan

Here we present the new phylogenetic tree manipulation class ‘TreeMan’ (see Fig. 1 for an overview); this is presented as the R package ‘treeman’ (N.B. the package name is all lowercase). This class is built around a list of named nodes rather than an index-based edge matrix as is the case for the phylo class. Using an edge matrix, whenever a node is added or removed the new positions of all nodes in the matrix must be determined and the tree must be re-computed. With a node list, however, order does not matter; nodes can be added and removed without altering the entire tree structure. Manipulations are also less dependent on tree size because all that is required is to update the local nodes: those that directly descend or ascend from the new node, converting that scale of computation time from *O(N* ^*2*^ *) to O(N)* (see Fig. 2 for a comparison of growing a tree with the phylo and TreeMan classes). Furthermore, with a node list the nodes in the tree can have unique IDs, which persist after insertions or deletions, allowing elements in a tree (such as node labels) to be more easily tracked during analysis. The subsequent sections of the paper describe the overall structure of the new class, describe treeman’s naming convention, and provide examples of tree manipulations that use the new package. The aims of treeman are to be conceptually intuitive for tree manipulation and as computationally efficient as possible within the R environment.

## Implementation

The TreeMan object in R is an S4 formal class whose main data slot is a list—which in R is a vector whose elements can be named. All nodes in a TreeMan object are named elements in this list (ndlst). Each node usually contains the following data slots: the node ID (id), the length of the preceding edge (spn, for “span”), the IDs of all connecting ascending/ancestral nodes to root (pre-node IDs, prid), the IDs of the immediately descending nodes (post-node IDs, ptid), and the IDs of all descending tips (kids). Additionally, if all nodes in a tree contain the spn slot, then each node will also contain: the total edge length of all descending nodes (phylogenetic diversity, pd), total edge length of all connected pre-nodes (prdst; in a rooted tree this is the root-to-tip distance), and the relative distance of the node in the tree (age, for a time-calibrated rooted tree). All nodes must have either a prid and/or ptid data slots: tip nodes have only prid slots, root nodes have only ptid slots, and internal nodes have both. These slots must contain IDs that are found within the ndlst; if they do not, an error is raised. These core slots are supplemented by optional slots, a non-unique taxonomic name that can be used to generate lineages (txnym) and user-defined slots that can contain any kind of information. In addition to the ndlst, the TreeMan object contains informative slots that are generated upon reading or generating the tree, and are updated whenever modified. Basic tree information can be seen by printing the tree to console.

The treeman package implements an intuitive naming convention in which each tree feature has a specific name that all methods and objects must use (see Fig. 1; Table 1). Specific tree or node information can be accessed using R indexing by character-string. For example, entering tree [“tips”] will output data on a tree’s tips. Double square brackets are used for pulling out information on individual nodes, e.g. tree [[“t1”]] will return node information on tip/node t1. The majority of methods in the treeman package are grouped into four main groups: *get*, *set*, *calc* and *manip* (see Table 1). The *get* methods return node or tree specific information, *set* methods change the tree’s or its nodes’ parameters, *calc* methods generate tree statistics, and *manip* methods alter the tree, usually by adding or removing tips and nodes. Methods that act across nodes are indicated with ‘nd’ or ‘nds’ in their function name, e.g. getNdAge() for a single node and getNdsAge() for multiple nodes. These core methods are designed to be fast and modular, allowing them to be readily combined into more complex functions. For example, evolutionary distinctness using the Fair Proportion metric [6] can be calculated by using the method calcFrPrp(). In implementation, this method uses getNdPrid() to get all pre-node IDs, runs getNdsKids() on these IDs to find the number of descendants per pre-node, and then sums the division of the numbers of descendants over the pre-nodes’ spans.

**Table 1:** Taxonomy of treeman functions

Method set	Description	Examples
*get*	Retrieve specific information about parts of a tree, often nodes	getNdAge, getPrnt, getNdKids, getNdLng, getNdPrid, getNdPtid, getPath, getSubtree
*calc*	Calculate tree statistics and tree associated information	calcDstMtrx, calcTrDst, calcPhyDv, calcFrPrp
*set*	Set node or overall tree values	setNdSpn, setAge, setPD, setRoot, setTol
*manip*	Change tree structure by adding or removing tips and nodes	addTip, rmTip, pinTip

Because the TreeMan class depends on the ndlst, all functions that run over this list are vectorised. All treeman functions that can be vectorised are done so using plyr vectorisation [11], providing substantial performance benefits, as computation is no longer taking place at the scripting level. Through the use of plyr these functions can also be parallelised using the “.parallel” argument that is passed onto plyr functions, which work in conjunction with parallel R packages such as DoMC [12] and doSNOW [13].

## Results and discussion

To demonstrate the TreeMan class and how the treeman functions can be combined to complete complex tasks, we demonstrate three use-cases: pinning missing taxa using online taxonomic databases to a molecular phylogenetic tree; simulating phylogenetic trees through time using different models of evolution; and testing for significant phylogenetic turnover between ecological communities.

### Tip pinning: adding missing taxa to a tree using online taxonomies

**Fig. 3:**
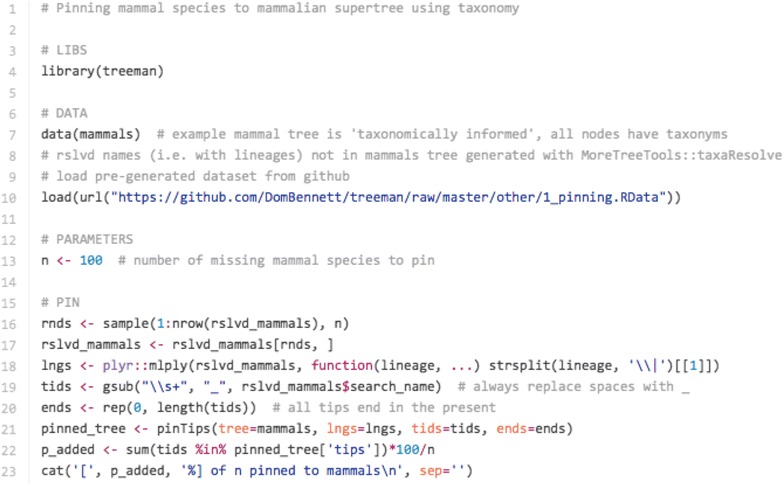
Code snippet used to generated “pinned” tree: load mammal supertree that comes with the treeman package, load resolved names of missing taxa pre-generated with MoreTreeTools, select 100 names at random, pin using pinTips()

**Fig. 4:**
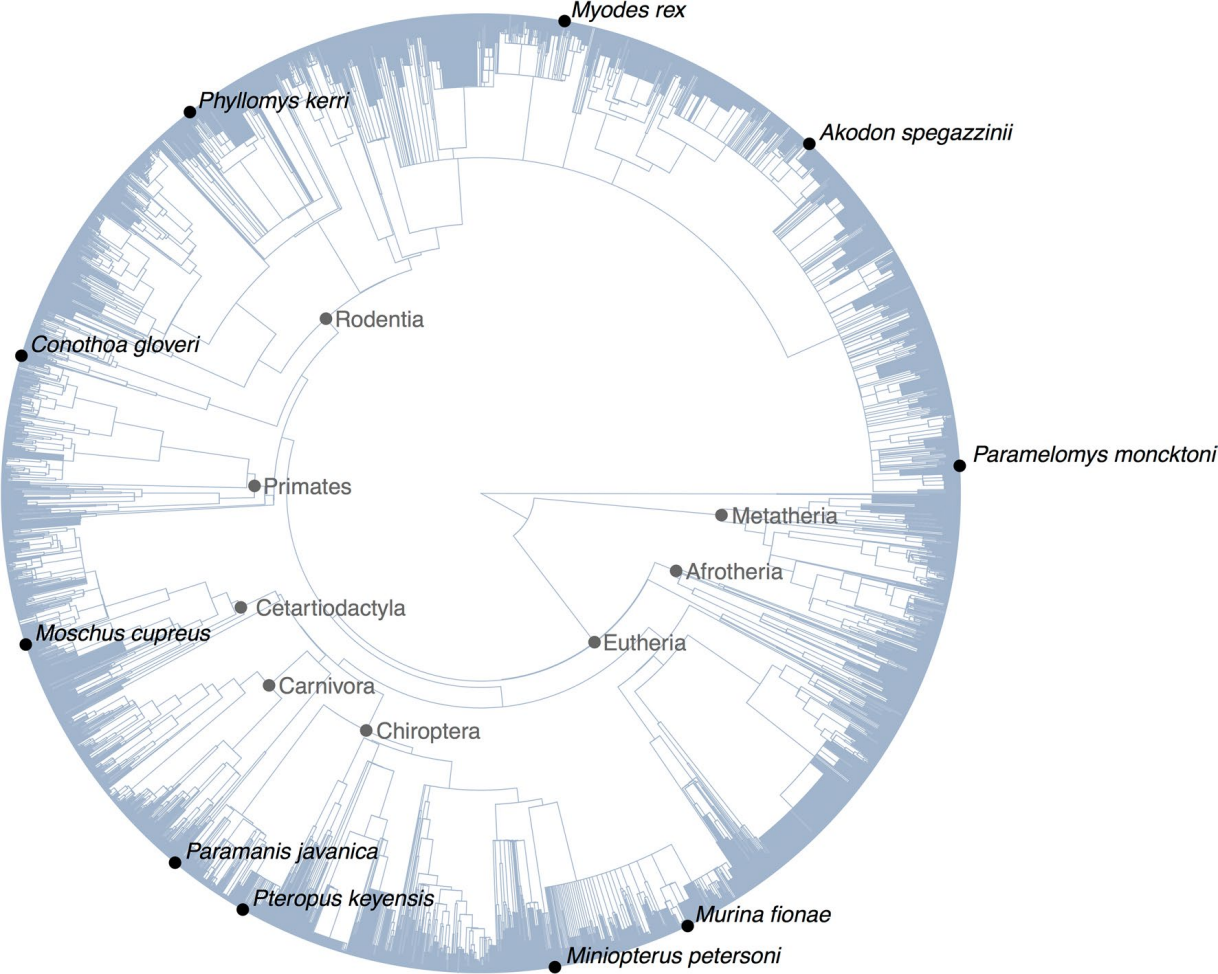
Mammalian supertree [15] showing ten tips that have been “pinned” using taxonomically constrained random placement

Trees often have missing tips due to a lack of data for phylogenetic construction. One approach to placing these tips is to use the taxonomy of the missing taxa and constraining placement with a model of evolution, as implemented by the Pastis software package [14]. Similar methods can be implemented in R using treeman. Our package provides an addTip() function that takes as arguments the incipient edge and an age range. Furthermore, TreeMan objects can be taxonomically informed: taxonomic names (txnyms) can be assigned to every node in a tree, allowing a user to constrain random placement of nodes. To demonstrate this functionality we present the mammalian supertree [15] that has been taxonomically informed using NCBI’s taxonomy [16]. We retrieved all the species names listed in NCBI but not present in the supertree, and pinned an example set of 100 new tips to the supertree. New tips were added to the supertree at any point in the branches that shared the lowest matching taxonomic rank with the taxonomy of the new tip (Figs. 3, 4). This was implemented with pinTips(), a function of 49 lines. The equivalent function using a phylo object is approximately 500 lines (see ‘pinning-with-phylo.R’ in Additional file 1).

### Tree simulation: generating trees using different models of evolution

**Fig. 5:**
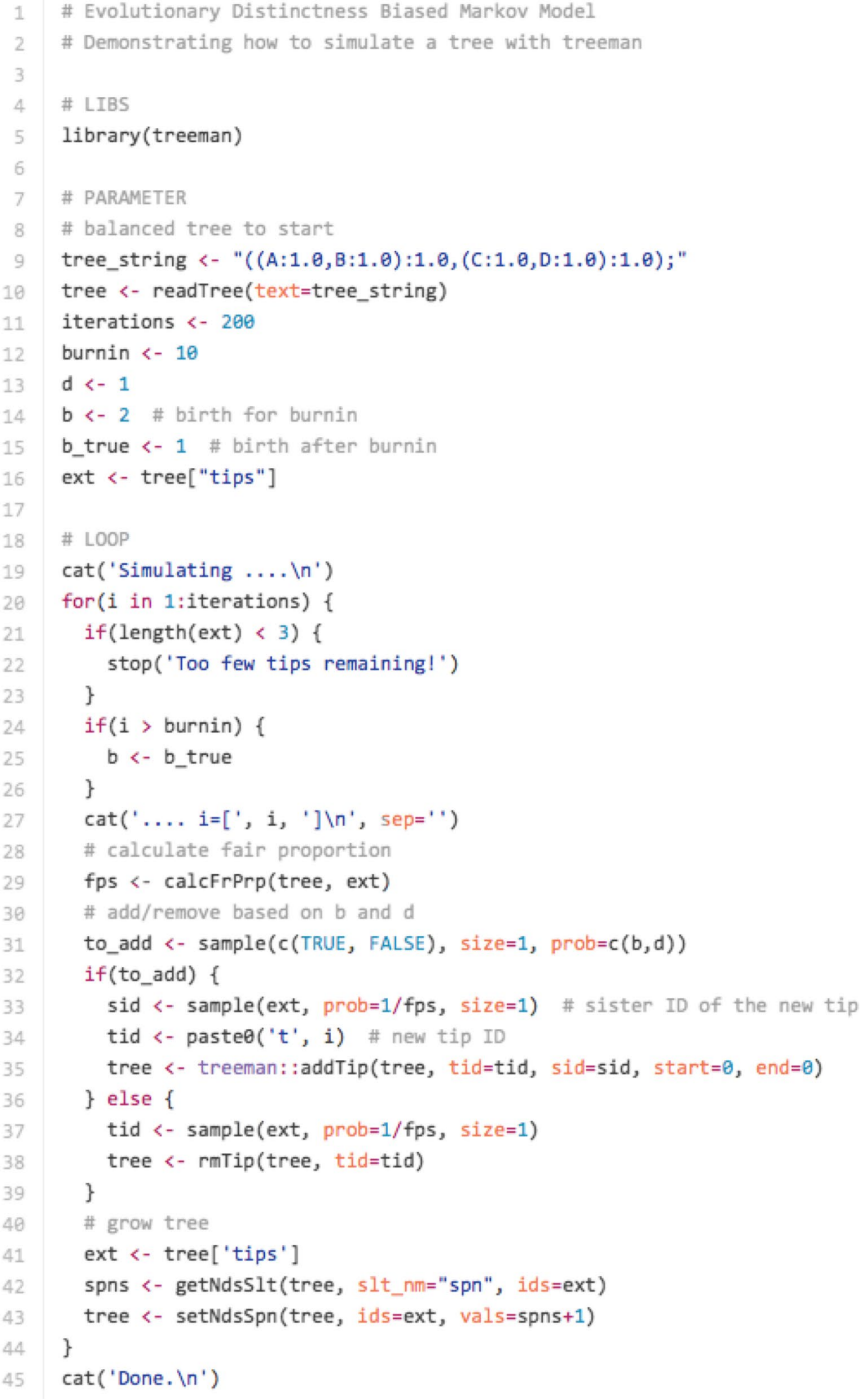
Code snippet for running EDBMM with treeman: read in balanced tree and set parameters for simulation; in loop, calculate fair proportion measure of evolutionary distinctness and add or remove tips based on these values; finally, extend the tip edges by adding 1

**Fig. 6:**
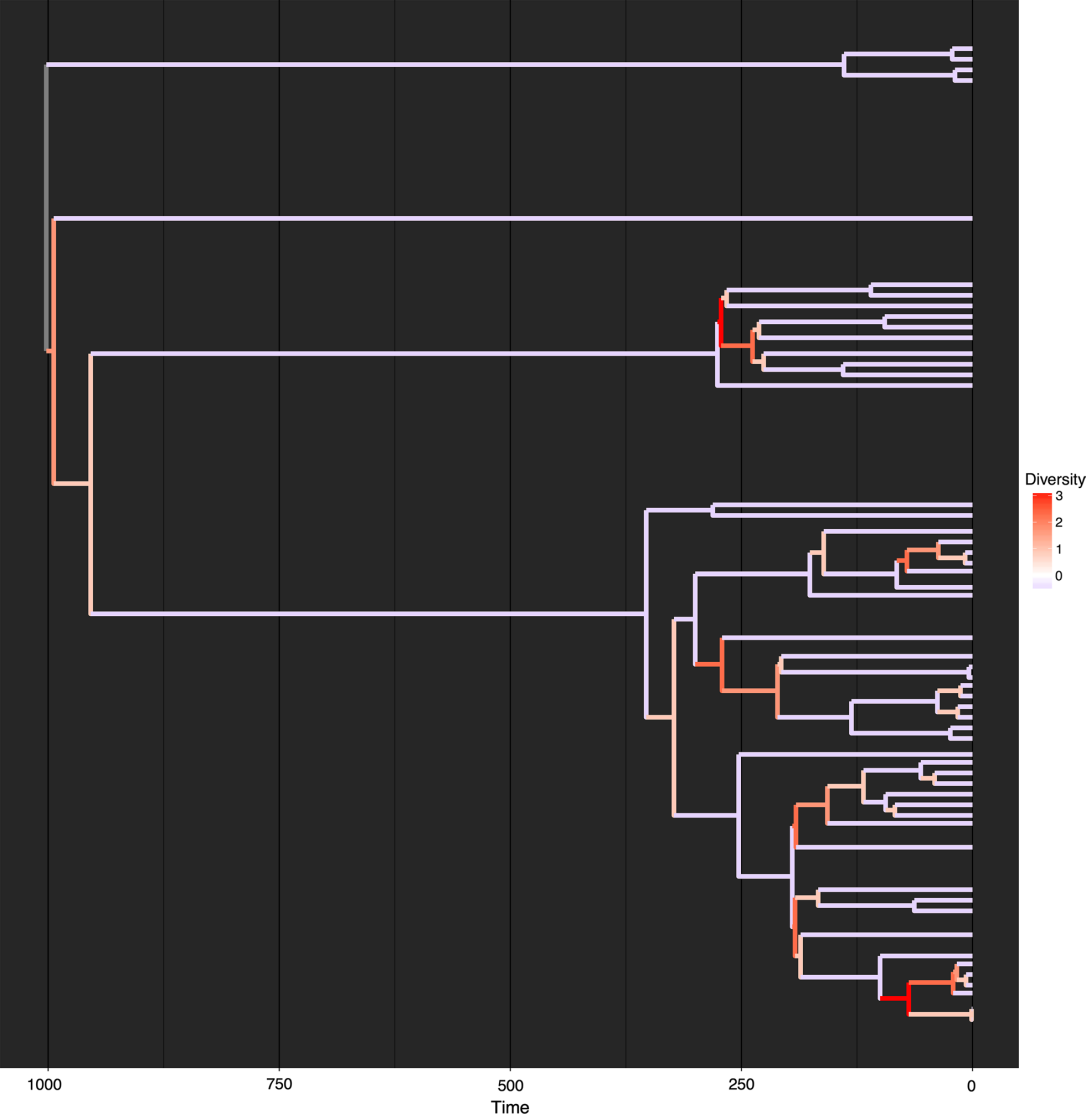
Phylogenetic tree simulated using an Evolutionary Distinctness Biased Markov Model (EDBMM), where tips with greater ED have lower rates of speciation and extinction. *Branch colours* indicate the local number of descending branches, in this case the number of descendants within 20 branch length units

Tree simulation is an important tool for exploring the processes that may have generated biodiversity. A common tool for simulating trees is the birth–death simulation or equals-rates Markov model (ERMM) that randomly removes and adds tips on a tree [17]. Many publications have independently explored alterations of the ERMM, such as modifying the rates of speciation for different tips in the tree [18–21]. All such studies, however, have developed their own software tools for tree simulation. The TreeMan object is easily modified using the addTip() and rmTip() functions and its speed of processing and implementation makes TreeMan an ideal software tool for tree simulation. Furthermore, as part of the R environment a user has access to the wide range of eco-evolutionary R packages already available to expand on these earlier tree simulations. To demonstrate this functionality, we produced an R script for simulating a tree using an Evolutionary Distinctness Biased Markov Model (EDBMM [21]) with fewer than 40 lines of R code (Fig. 5). The simulation randomly adds and removes tips but with a bias towards evolutionary distinctness: more evolutionarily distinct tips have a lower rate of speciation and extinction (Fig. 6). The equivalent script for running a vectorised EDBMM with a phylo object takes 188 lines (see ‘edbmm-with-phylo.R’ in Additional file 2).

### Testing for significant phylogenetic turnover

**Fig. 7:**
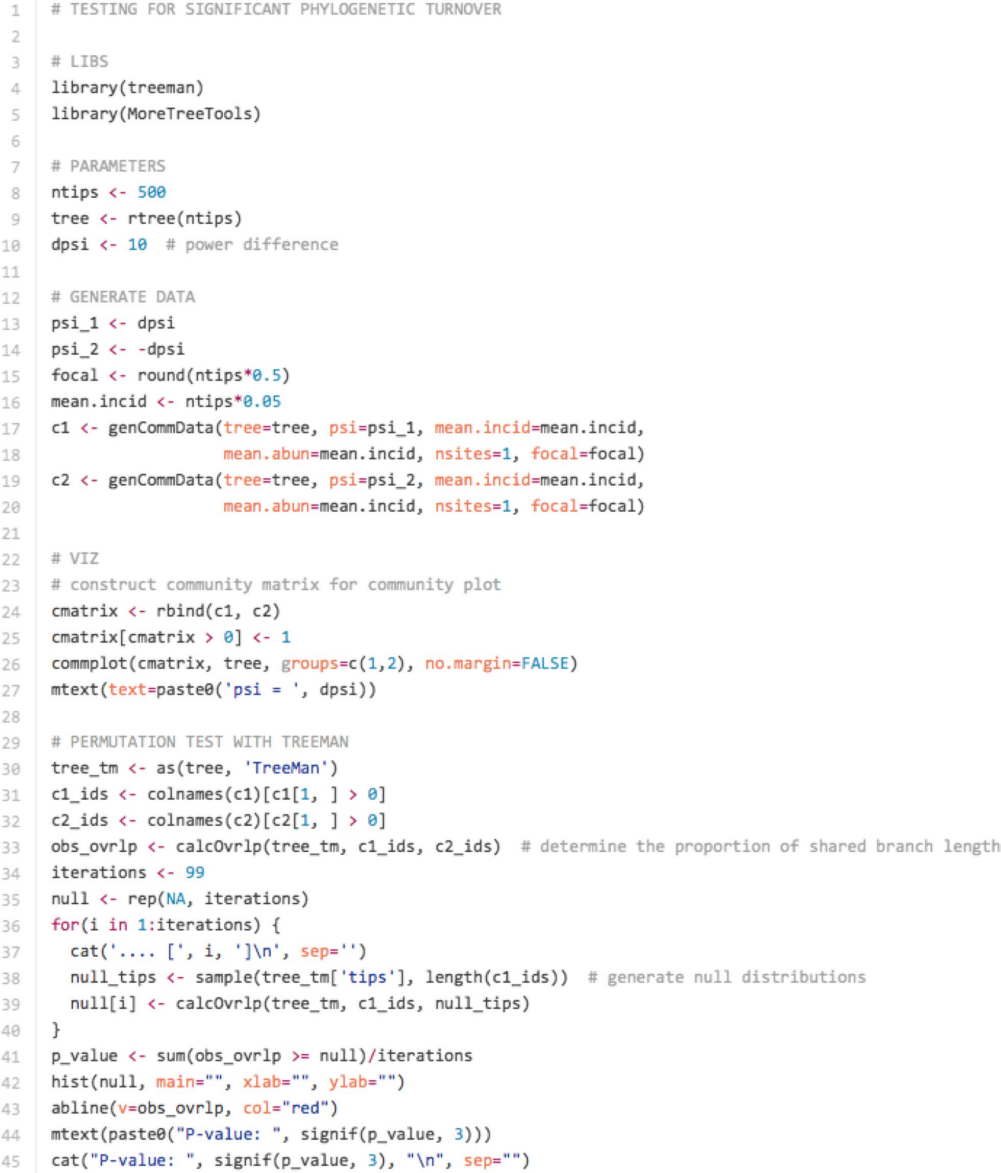
Code snippet for calculating overlap between two different communities using treeman and MoreTreeTool: generate random communities using parameters of community overlap for a random tree, plot as community trees (see Fig. 8), convert trees from phylo to TreeMan using MoreTreeTool [24] ’s as(), generate null communities to test whether the two communities have significant overlap

**Fig. 8:**
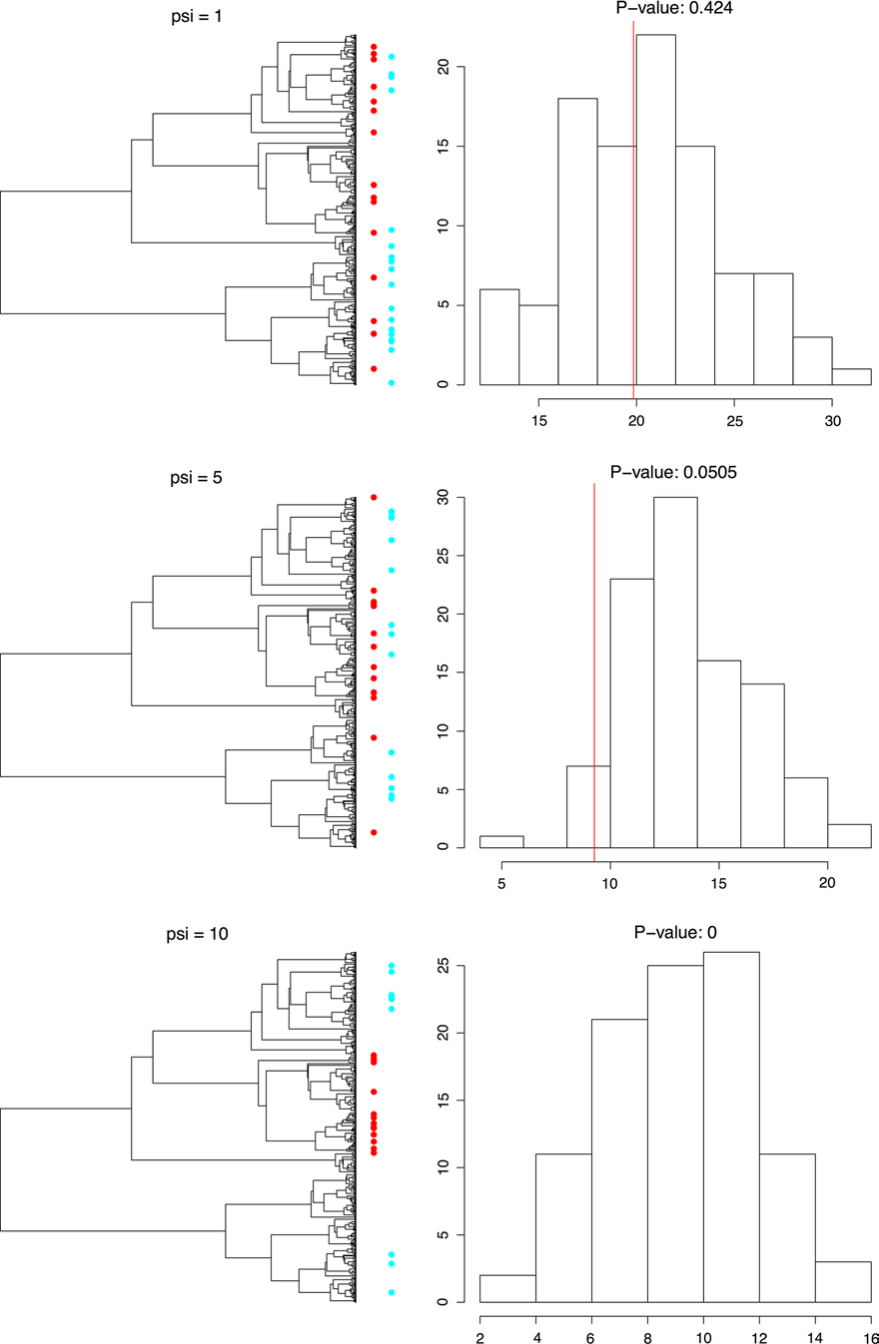
Using calcOvrlp() to test for phylogenetic turnover. *Left*, phylogenetic trees with distributions of taxa found in two non-overlapping communities (*red* and *blue*) randomly generated with phylogenetic distance bias. The strength of this bias is determined by psi; greater psi leads to reduced overlap between communities. *Right*, distribution of 99 iterations for null communities’ overlap in phylogenetic diversity with *red community*. *Red line* indicates observed overlap between *red* and *blue communities*

A common need in biological analysis is the detection of phylogenetic signal. Such analyses are often executed using model-based statistical analyses for which APE has been primarily designed [1]. A model-based approach, however, is not always applicable to every question related to phylogenetic signal. One such question is whether the changes in species between habitat types are due to phylogenetic signal in species’ gains and losses, e.g. as a result of human-caused habitat loss [22]. A useful metric for calculating such a difference is the UniFrac measure [23], which measures the unique and shared fractions of branches represented between communities. The TreeMan object is well suited to calculating this metric as all nodes in a tree have IDs; even if nodes are added or removed, IDs are constant. To demonstrate this we randomly generated community data with different intensities of overlap. We then ran permutation tests that detect whether there has been significant phylogenetic turnover between these communities using treeman’s calcOvrlp() (Figs. 7, 8).

## Conclusions

TreeMan is an S4 class that encodes a phylogenetic tree using a node list. The advantage of a node list is the faster computational processing, and the ready capacity to track nodes between manipulations and vectorise or parallelise large-scale tree manipulations. The treeman package introduces new terminology to describe different elements of a tree and uses a naming convention to combine these new terms to make a more intuitive set of methods for tree manipulation. 

## Additional files

**Additional file 1.** Pinning-with-phylo.R.

**Additional file 2.** Edbmm with-phylo.R.
